# *In Vivo* Indicators of Cytoplasmic, Vacuolar, and Extracellular pH Using pHluorin2 in *Candida albicans*

**DOI:** 10.1128/mSphere.00276-17

**Published:** 2017-07-05

**Authors:** Hélène Tournu, Arturo Luna-Tapia, Brian M. Peters, Glen E. Palmer

**Affiliations:** Department of Clinical Pharmacy, College of Pharmacy, University of Tennessee Health Sciences Center, Memphis, Tennessee, USA; Duke University Medical Center

**Keywords:** *Candida albicans*, chemical screening, pH dynamics, vacuoles

## Abstract

Environmental or chemically induced stresses often trigger physiological responses that regulate intracellular pH. As such, the capacity to detect pH changes in real time and within live cells is of fundamental importance to essentially all aspects of biology. In this respect, pHluorin, a pH-sensitive variant of green fluorescent protein, has provided an invaluable tool to detect such responses. Here, we report the adaptation of pHluorin2 (PHL2), a substantially brighter variant of pHluorin, for use with the human fungal pathogen *Candida albicans*. As well as a cytoplasmic PHL2 indicator, we describe a version that specifically localizes within the fungal vacuole, an acidified subcellular compartment with important functions in nutrient storage and pH homeostasis. In addition, by means of a glycophosphatidylinositol-anchored PHL2-fusion protein, we generated a cell surface pH sensor. We demonstrated the utility of these tools in several applications, including accurate intracellular and extracellular pH measurements in individual cells via flow cytometry and in cell populations via a convenient plate reader-based protocol. The PHL2 tools can also be used for endpoint as well as time course experiments and to conduct chemical screens to identify drugs that alter normal pH homeostasis. These tools enable observation of the highly dynamic intracellular pH shifts that occur throughout the fungal growth cycle, as well as in response to various chemical treatments.

**IMPORTANCE** *Candida albicans* is an opportunistic fungal pathogen that colonizes the reproductive and gastrointestinal tracts of its human host. It can also invade the bloodstream and deeper organs of immunosuppressed individuals, and thus it encounters enormous variations in external pH *in vivo*. Accordingly, survival within such diverse niches necessitates robust adaptive responses to regulate intracellular pH. However, the impact of antifungal drugs upon these adaptive responses, and on intracellular pH in general, is not well characterized. Furthermore, the tools and methods currently available to directly monitor intracellular pH in *C. albicans*, as well as other fungal pathogens, have significant limitations. To address these issues, we developed a new and improved set of pH sensors based on the pH-responsive fluorescent protein pHluorin. This includes a cytoplasmic sensor, a probe that localizes inside the fungal vacuole (an acidified compartment that plays a central role in intracellular pH homeostasis), and a cell surface probe that can detect changes in extracellular pH. These tools can be used to monitor pH within single *C. albicans* cells or in cell populations in real time through convenient and high-throughput assays.

## Abstract (precis)

*Candida albicans* is an opportunistic fungal pathogen that colonizes the reproductive and gastrointestinal tracts of its human host. It can also invade the bloodstream and deeper organs of immunosuppressed individuals, and thus it encounters enormous variations in external pH *in vivo*. Accordingly, survival within such diverse niches necessitates robust adaptive responses to regulate intracellular pH. However, the impact of antifungal drugs upon these adaptive responses, and on intracellular pH in general, is not well characterized. Furthermore, the tools and methods currently available to directly monitor intracellular pH in *C. albicans*, as well as other fungal pathogens, have significant limitations. To address these issues, we developed a new and improved set of pH sensors based on the pH-responsive fluorescent protein pHluorin. This includes a cytoplasmic sensor, a probe that localizes inside the fungal vacuole (an acidified compartment that plays a central role in intracellular pH homeostasis), and a cell surface probe that can detect changes in extracellular pH. These tools can be used to monitor pH within single *C. albicans* cells or in cell populations in real time through convenient and high-throughput assays.

## INTRODUCTION

The pH-sensitive fluorescent protein pHluorin (PHL) has been widely applied to measure cytoplasmic pH in live cells of many species (1, 2), including recently the human fungal pathogen *Candida albicans* (3, 4). PHL is a variant of green fluorescent protein (GFP) with a bimodal excitation spectrum that is pH dependent. Following excitation at 395 or 475 nm, fluorescence emission is measured at 509 nm, and the ratio of the two measurements is used to estimate the proximal pH by means of a calibration curve. While PHL-based probes have been broadly adopted to measure cytoplasmic pH, to date they have not been widely used to measure physiological pH within the fungal vacuole, an acidified organelle. Several studies in the model yeast *Saccharomyces cerevisiae* utilized an “ecliptic” variant of PHL which loses fluorescence under acidic conditions, to assess pH changes during transport through the endosomal network to the fungal vacuole (5, 6). These measurements are based upon microscopic observation and quantification of signal intensity at single excitation/emission wavelengths, with PHL fluorescence quenched upon reaching the acidified vacuolar lumen. However, analyses with ecliptic versions of PHL can be confounded by variations in the expression level, stability, or proteolysis of the PHL probe itself between strains, growth conditions, or chemical treatments (7, 8). As such, for many applications, ratiometric variants of PHL are preferred, as they are largely insensitive to variable expression levels, stability, or proteolysis.

The current gold standard for measuring pH within the vacuolar lumen includes the pH-sensitive dye 2′,7′-bis(2-carboxyethyl)-5(and-6)-carboxyfluorescein (BCECF) (9, 10). However, BCECF-based protocols require multiple manipulations of large culture volumes and are therefore incompatible with real-time measurements in individual cells or populations of cells *in situ*. The intracellular accumulation of the acidotrophic base quinacrine within the vacuole can provide a more convenient surrogate marker of vacuolar acidification (11), but it is at best a qualitative measure and its reliability has been called into question. To address these limitations, we set out to develop a new ratiometric PHL-based probe that could facilitate rapid and convenient measurements of intravacuolar pH in *C. albicans*.

A recent study reported a new variant of ratiometric PHL, pHluorin2 (PHL2), with improved folding efficiency and brightness (7). Here, we report the adaptation of PHL2 for use in *C. albicans* and the first pHluorin-based sensor of vacuolar pH. We also describe a cell surface-localized PHL2 fusion protein that can be used to detect changes in extracellular pH. The PHL2-based set of probes (cytoplasmic, vacuolar, and cell surface) facilitates rapid measurement of intracellular and extracellular pH of cell populations by using a convenient 96-well plate-based format or in individual cells by using flow cytometry.

## RESULTS

### *Candida albicans*-optimized PHL2 as a cytosolic and vacuolar pH probe.

**FIG 1:**
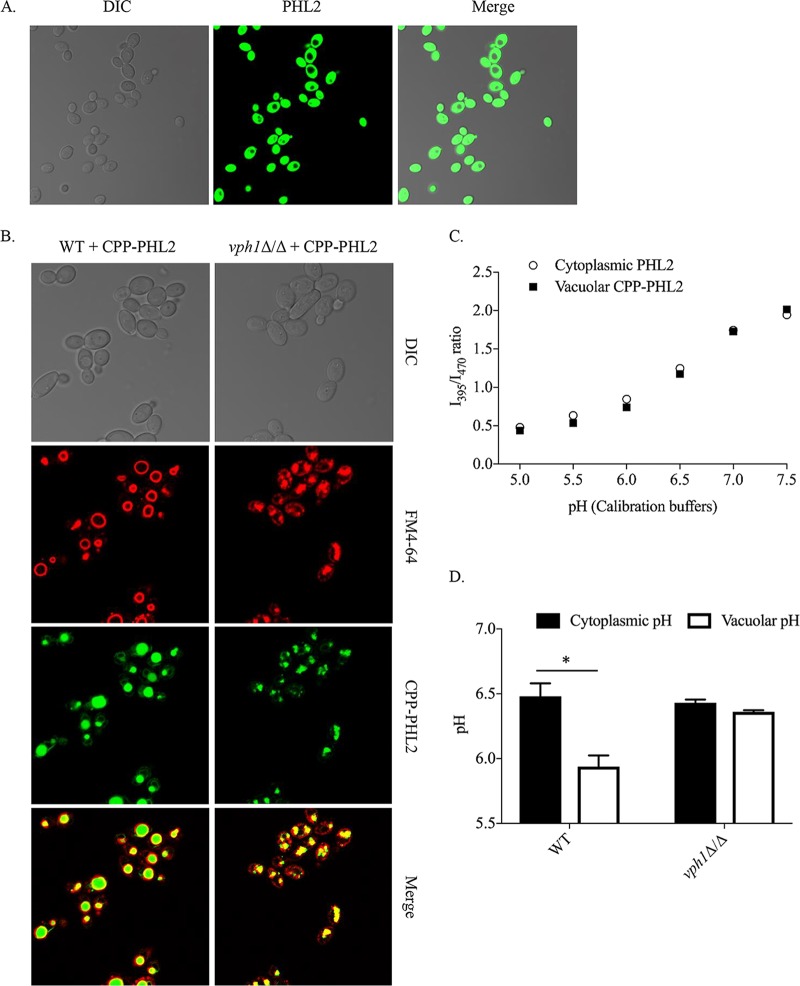
*C. albicans*-optimized PHL2 and CPP-PHL2 probes localize to the cytoplasm and vacuole, respectively. (A) The *P*_*TEF1*_-*PHL2* construct was introduced into wild-type strain CAI4, and PHL2 fluorescence was observed by confocal microscopy. (B) The *P_ACT1_-CPP-PHL2* expression construct was introduced into CAI4 (WT) (left panels) or the VATPase-deficient *vph1Δ/Δ* mutant (right panels). Cells were grown in minimal medium and preincubated with FM4-64 to label the vacuolar membrane. Images were then acquired by confocal microscopy using differential interference contrast optics and 488-nm and 546-nm lasers for PHL2 and FM4-64, respectively. (C) CAI4 cells expressing PHL2 or CPP-PHL2 were resuspended in precalibrated buffers (containing 10 mM sodium azide) at the indicated pH. Fluorescence emission intensity was then measured at a fixed wavelength of 509 nm (9-nm bandwidth), following excitation at 395 (I_395_) and 470 nm (I_470_) (9-nm bandwidth), by using a monochromator-based plate reader. The I_395_/I_470_ ratio was calculated and plotted against the buffer’s pH to generate a calibration curve. (D) CAI4 (WT) and *vph1Δ/Δ* strains expressing PHL2 (cytoplasmic) or CPP-PHL2 (vacuolar) were grown in unbuffered minimal medium, and the I_395_/I_470_ ratios of intact cells were determined as described for panel C, except without permeabilization. Cytoplasmic and vacuolar pHs were then estimated for each strain by using a third-degree polynomial regression equation based on each strain’s calibration curve. Means and standard deviations were calculated from four biological replicates for each strain. *, *P* < 0.0001.

The coding sequence of PHL2 (7) was codon optimized for efficient translation in *C. albicans* (see Table S1 in the supplemental material) and cloned downstream of the strong *TEF1* promoter (*P*_*TEF1*_) in our pKE4 expression vector. Following introduction into *C. albicans*, high levels of cytoplasmic PHL2-derived fluorescence were observed, as expected (Fig. 1A). A vacuole-targeted version of PHL2 was also produced through fusion to the C terminus of the *C. albicans* carboxypeptidase Y (Cpy1p) prepropeptide (codons 1 to 129) (12, 13) to yield CPP-PHL2. The CPP-PHL2 fusion was placed under the transcriptional control of the less powerful *ACT1* promoter (*P*_*ACT1*_) to avoid the risk of overexpression-induced mislocalization resulting from saturation of the CPY sorting receptor (14). Following introduction of the CPP-PHL2 expression construct into *C. albicans*, PHL2 fluorescence was localized specifically inside vacuoles, as illustrated by colabeling with the vacuolar membrane dye FM4-64 (Fig. 1B). The pH-dependent bimodal fluorescence of cytoplasmic and vacuolar PHL2 probes was tested by permeabilizing PHL2- and CPP-PHL2-expressing *C. albicans* cells in buffers with a wide range of physiologically relevant pHs. Both probes demonstrated the expected bimodal excitation and pH-dependent responses (Fig. S1A), with the I_395_/I_470_ ratios (ratio of fluorescence emission at 509 nm following excitation at either 395 or 470 nm) obtained from both PHL2 and CPP-PHL2 calibration curves essentially identical (Fig. 1C). While these data were obtained from cell pellets via a plate reader, similar pH-dependent responses were obtained from single cells expressing PHL2 or CPP-PHL2 when measured by flow cytometry (Fig. S1B). Background fluorescence was measured with a strain carrying vector alone (no PHL2 expression). PHL2-derived signal-to-background fluorescence ratios were high at both excitation wavelengths based on both detection methods (Fig. S1C), indicating that background fluorescence did not complicate analysis.

10.1128/mSphere.00276-17.1 FIG S1  Spectral emission, calibration, and background of the intracellular probes. (A) Fluorescence emission of vacuolar PHL2. WT (left) and *vph1Δ/Δ* mutant (right) strains expressing the CPP-PHL2 probe were grown in minimal medium, washed, permeabilized, and resuspended in precalibrated buffers at the indicated pH. Cell suspensions were then transferred to a 96-well plate, and spectral scans were performed over a range of excitation wavelengths (9-nm bandwidth), with fluorescence emission measured at a fixed wavelength of 509 nm (9-nm bandwidth), using a monochromator-based plate reader. RFU, relative fluorescence units. (B) Cell population versus single-cell fluorescence measurements. Wild-type cells expressing PHL2 (left) or CPP-PHL2 (right) were grown overnight (O/N) in minimal medium, and approximately 10^7^ cells were transferred to calibration buffers prepared at the indicated pHs. Using the plate reader, samples were then transferred to 96-well plate and pelleted prior to fluorescence measurements at 395 nm and 470 nm. The I_395_/I_470_ ratios were then calculated and plotted against each individual pH (full circles and triangles). By flow cytometry, 50,000 events were counted and the median fluorescence levels at 405 nm and 488 nm were determined (empty circles and triangles). (C) Background fluorescence via both detection methods. Cells expressing PHL2 were calibrated in buffers at the indicated pHs, and the same strain expressing the empty vector was used to control for background fluorescence at the measured wavelengths. Flow cytometry-derived median fluorescence levels at 405 and 488 nm are presented as stacked RFU for both wavelengths (left). Cells expressing CPP-PHL2 were calibrated at the indicated pHs. The empty vector-expressing strain was also calibrated in the same buffers for background fluorescence assessment. Stacked RFU data at 395 nm and 470 nm (fixed excitation at 509 nm) from pelleted cells were obtained via a monochromator-based plate reader (right panel). Download FIG S1, PDF file, 0.1 MB. Copyright © 2017 Tournu et al. 2017 Tournu et al. This content is distributed under the terms of the Creative Commons Attribution 4.0 International license.

10.1128/mSphere.00276-17.5 TABLE S1  Primers used in this study and the synthesized *Candida albicans*-optimized PHL2 sequence. Download TABLE S1, PDF file, 0.01 MB. Copyright © 2017 Tournu et al. 2017 Tournu et al. This content is distributed under the terms of the Creative Commons Attribution 4.0 International license.

We further validated the PHL2 probes by introducing them into a *C. albicans vph1Δ/Δ* mutant which lacks a subunit of the V-ATPase proton pump that is responsible for vacuolar acidification (9, 10, 15). Despite the aberrant vacuolar morphology of the *vph1Δ/Δ* mutant, CPP-PHL2 correctly localized within FM4-64-labeled vacuoles (Fig. 1B). Calibration experiments confirmed that CPP-PHL2 is fully functional when expressed in the *vph1Δ/Δ* mutant, undergoing pH-dependent shifts in I_395_/I_470_ ratios that are indistinguishable from the wild-type (WT) response (Fig. S1A). Following culture in yeast nitrogen base (YNB) medium, significantly higher I_395_/I_470_ ratios were measured with the CPP-PHL2 probe in the *vph1Δ/Δ* mutant than in the wild type, indicating an elevated vacuolar pH in the mutant (Fig. 1D). As expected, the mutant and wild-type strains had similar I_395_/I_470_ ratios with the PHL2 probe, indicating that cytoplasmic pH is unaffected in the *vph1Δ/Δ* mutant.

### A cell surface PHL2 probe can detect changes in extracellular pH.

**FIG 2:**
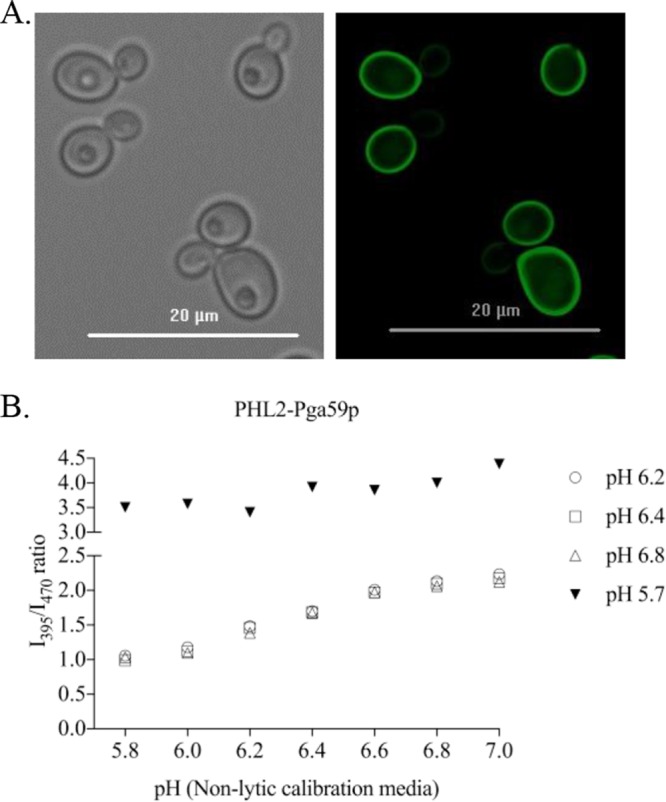
PHL2-Pga59p fusion provides a cell surface pH sensor for *C. albicans*. (A) CAI4 cells expressing the PHL2-Pga59p fusion protein were grown in minimal medium at pH 7. Bright-field (right panel) and fluorescent images (enhanced GFP filter at 485 nm [20-nm bandwidth]) (left panel) were acquired at 60× magnification using an epifluorescence microscope. (B) CAI4 cells expressing PHL2-Pga59p were grown overnight in minimal medium buffered at pH 6.2, 6.4, and 6.8 (open symbols) and at pH 5.7 and transferred to nonlytic medium prepared at the indicated pHs. Fluorescence intensity was then measured at a fixed wavelength of 509 nm (9-nm bandwidth), following excitation at 395 (I_395_) and 470 nm (I_470_) (9-nm bandwidth) using a monochromator-based plate reader. The I_395_/I_470_ ratio was calculated and plotted against the buffer’s pH to generate a calibration curve.

We expanded the pH sensor toolbox by creating a cell surface-bound PHL2 probe as a fusion with the previously described glycophosphatidylinositol (GPI)-anchored Pga59 protein (16). The PHL2-Pga59p fusion protein localized brightly at the cell surface (Fig. 2A). Furthermore, the PHL2-Pga59p fusion displayed pH-dependent spectral shifts similar to those of the cytoplasmic and vacuolar probes when calibrated under nonlytic conditions (Fig. 2B). Exposure of cells to media buffered at different pHs was sufficient to generate a calibration curve, validating the extracellular orientation of the PHL2 tag in the GPI-anchored fusion protein. Notably, PHL2-Pga59p cells did not retain their I_395_/I_470_ ratios following fixation (data not shown).

During calibration experiments with PHL2-Pga59p, the spectral shift occurred immediately after transfer into the calibration buffers over a wide range of pHs, from 5 to 7. However, PHL2-Pga59p fluorescence was lost when cells were grown for prolonged periods (>4 to 5 h) in medium with a pH of <6 (Fig. S2). A similar loss of fluorescence was observed with two additional cell surface PHL2 fusions produced with Pga62p (a cell wall GPI-anchored protein) and Sur7p, an integral membrane protein (data not shown). We hypothesized that the loss of fluorescence at pHs of <6 resulted from proteolysis of the PHL2-Pga59p fusion by secreted aspartyl proteases (SAPs), which are known to be differentially regulated by pH in *C. albicans* (17, 18). However, expression of the fusion in either the *sap1Δ/Δ sap2Δ/Δ sap3Δ/Δ* or *sap4Δ/Δ sap5Δ/Δ sap6Δ/Δ* triple mutant strain (19, 20) did not stabilize PHL2 fluorescence at the cell surface at low pH (data not shown). We also considered the possibility that the fusion protein may be released from the cell surface at lower pH. However, no fluorescence above background was detected in the supernatant of cell cultures grown at different pHs (data not shown). Ultimately, it seems that continuous exposure of PHL2 to an environment of pHs of <6 may quench or bleach the PHL2 chromophore. As such, the PHL2 probes can only be used to detect transient responses below pH 6. However, our capacity to reliably detect PHL2 within the vacuole, the most acidic compartment within the fungal cell, supports that this is unlikely to be a major limitation for intracellular pH measurements.

10.1128/mSphere.00276-17.2 FIG S2  Loss of fluorescence at pHs below 6. Cells expressing the PHL2-Pga59 fusion protein were grown overnight in media at the indicated pHs. Bright-field (right) and fluorescent images (enhanced GFP [eGFP] excitation filter of 485 nm [20-nm bandwidth]) (left) were acquired using a BioTek Synergy imaging reader. Magnification, ×60. The same iteration time and gain were used for all the cell cultures. Download FIG S2, PDF file, 0.1 MB. Copyright © 2017 Tournu et al. 2017 Tournu et al. This content is distributed under the terms of the Creative Commons Attribution 4.0 International license.

### PHL2-based probes facilitate noninvasive and real-time detection of physiological pH responses.

**FIG 3:**
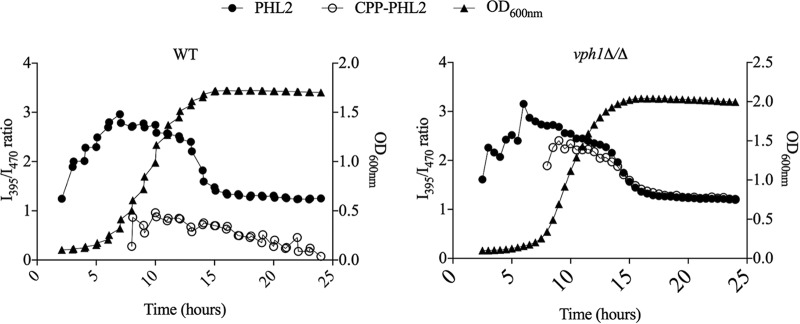
Intracellular pH varies with growth phase in *C. albicans***.** CAI4 (WT) (left panel) and *vph1Δ/Δ* mutant (right panel) expressing PHL2 (full circles), or CPP-PHL2 (open squares) were grown in minimal medium in 96-well plates at 35°C for 24 h within a BioTek Cytation 5 plate reader. I395, I470, and OD600nm were measured every 30 min as described in Fig. 1. Background fluorescence at either wavelength was measured at the same intervals from a strain carrying the empty vector (minus PHL2 control), and subtracted from the total fluorescence intensity to yield PHL2-derived fluorescence at either wavelength. I395/I470 ration was then calculated for each time point and plotted against time.

We next performed time course experiments to monitor intracellular pH changes during the normal growth cycle of *C. albicans*. Cells expressing PHL2, CPP-PHL2, or vector alone (minus PHL2, to determine background fluorescence) were seeded into the wells of a 96-well plate in YNB medium and I_395_/I_470_ ratios, as well as the absorbance at 600 nm, were measured at 30-min intervals over a period of 24 h. We could reproducibly obtain data above background in the first few hours for the cytoplasmic probe. The vacuolar probe, being less bright, could be reproducibly monitored (above background) after 4 to 6 h of growth, when sufficient cell numbers were present. In wild-type cells, cytoplasmic pH rose as cells entered the exponential phase of growth (Fig. 3A). This was followed by a sharp drop in pH coinciding with growth arrest, with the cytoplasm remaining acidified throughout stationary phase. This result is in good agreement with a previous pHluorin-based study in the model yeast *S. cerevisiae* (21), in which acidification of the cytoplasm upon nutrient depletion was reported. The CPP-PHL2 probe indicated that intravacuolar pH was more acidic and less dynamic than cytoplasmic pH throughout the growth cycle. A broadly similar pattern of cytoplasmic pH change was observed through the growth cycle of the *vph1Δ/Δ* V-ATPase mutant. However, in contrast to the wild type, vacuolar pH was indistinguishable from cytoplasmic pH, consistent with the mutant’s inability to acidify the vacuolar lumen (Fig. 3B).

### Intravacuolar pH varies among clinical isolates.

**FIG 4:**
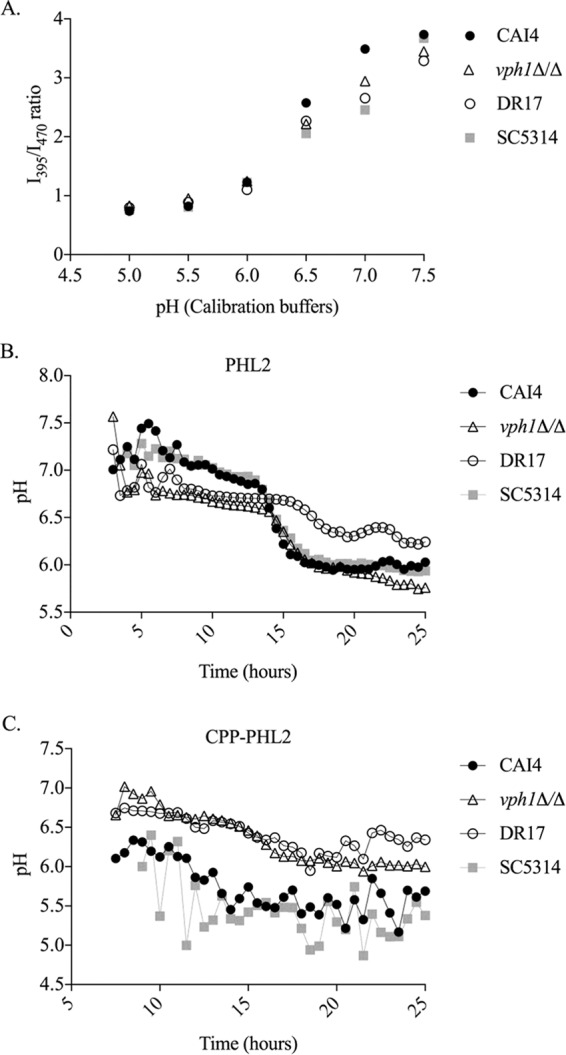
Cytoplasmic and vacuolar pHs vary among clinical isolates of *C. albicans***.** Cytoplasmic PHL2 and CPP-PHL2 expression constructs were introduced into CAI4 (filled circles), *vph1Δ/Δ* mutant strain (open triangles), the clinical isolate DR17 (open circles), and SC5314 (gray squares). (A) To generate calibration curves, cultures of each strain expressing CPPPHL2 were resuspended in precalibrated buffers at the indicated pH. The I_395_ and I_470_ was then measured using a monochromator-based plate reader as described for previous figures. The I_395_/I_470_ ratio was calculated and plotted against each buffer’s pH to generate the calibration curve. Strains expressing PHL2 (B) or CPP-PHL2 (C) were grown in minimal medium in a 96-well plate, and the I_395_/I_470_ ratios of intact cells as well as the OD_600_ were determined every 30 min as described for Fig. 3. For each strain, a third-degree polynomial regression equation based on the respective calibration curve was generated to calculate the cytoplasmic (B) and vacuolar (C) pH.

To facilitate the use of the PHL2 probes in a variety of prototrophic laboratory and clinical isolates of *C. albicans*, the PHL2 and CPP-PHL2 expression cassettes were transferred into pDUP3, a vector containing the *SAT1* selection marker that confers resistance to nourseothricin and which can be targeted for integration into the neutral *NEUT5L* locus (22). The resulting PHL2 and CPP-PHL2 expression vectors were introduced into a collection of clinical isolates, each of which has homozygous single nucleotide polymorphisms (SNPs) in vacuolar proton pump (V-ATPase)-related genes compared to the reference strain SC5314 (Dave Rogers, personal communication). Calibration curves were generated for each individual strain (Fig. 4A), and time course experiments in minimal medium revealed that one isolate (DR17) showed higher CPP-PHL2-derived I_395_/I_470_ ratios, indicating a higher vacuolar pH, than the widely used reference strains CAI4 and SC5314 (Fig. 4B). In fact the vacuolar pH measurements for isolate DR17 were strikingly similar to those of the *vph1Δ/Δ* mutant, suggesting DR17 could be deficient in V-ATPase activity. Cytoplasmic pH among the same isolates did not vary greatly, although DR17 displayed a less dramatic drop in cytoplasmic pH upon entry into stationary phase than the reference strains SC5314 and CAI4 (Fig. 4C). Collectively, these results suggest that DR17 has an abnormal intracellular pH homeostasis.

### A PHL2-based chemical screen identified drugs that disrupt *C. albicans* intracellular pH homeostasis.

**FIG 5:**
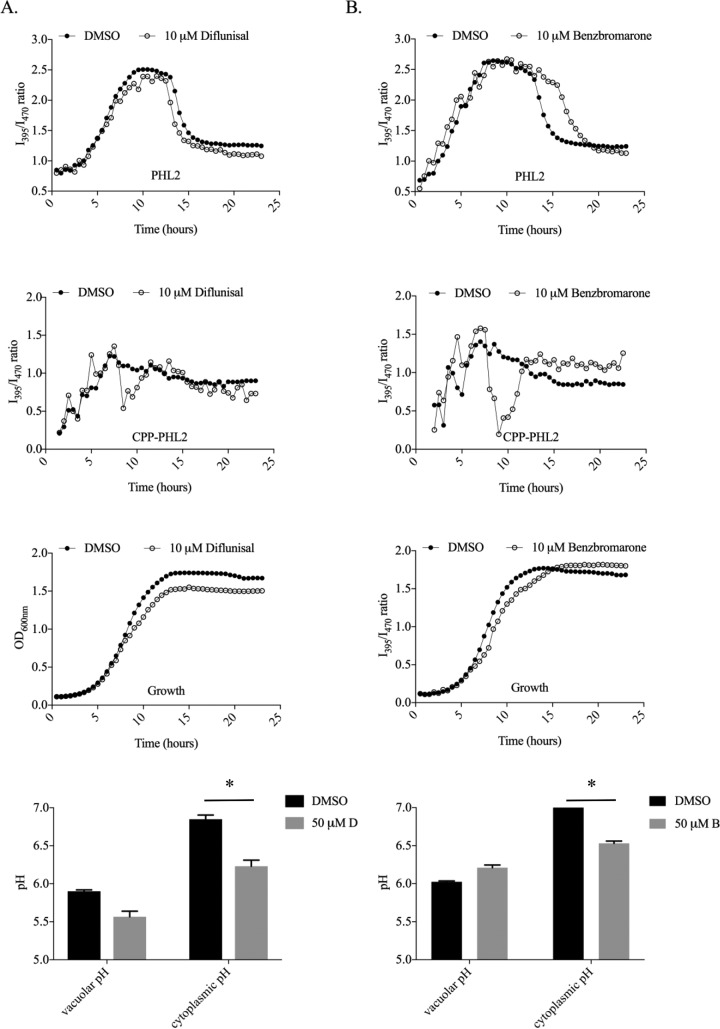
Diflunisal and Benzbromarone differentially affect cytoplasmic and vacuolar pHs in *C. albicans***.** PHL2- and CPP-PHL2-expressing strains of *C. albicans* (CAI4 strain background) were used to screen the NCC compound library in time course assays as described for Fig. 3, with each compound at a final concentration of 10 μM. No-drug control wells contained an equivalent amount of the DMSO vehicle alone. Growth (the OD_600_) and fluorescence intensity was measured every 30 min, and I_395_/I_470_ ratios were calculated for each time point after background fluorescence (measured from a strain carrying the empty expression vector) was subtracted. Data for two compounds in the collection, diflunisal (A) and Benzbromarone (B), are shown. In a separate follow-up experiment (bottom graphs in both panels A and B), endpoint measurements were taken from the PHL2- and CPP-PHL2-expressing strains grown for 24 h at 30°C in minimal medium plus 50 mM diflunisal (labeled 50 mM in panel D) or Benzbromarone (labeled 50 mM in panel B). Growth (the OD_600_) and fluorescence intensity were measured as described above, and cytoplasmic as well as vacuolar pHs were estimated from the respective I_395_/I_470_ ratios by using a third-degree polynomial regression equation based on each strain’s respective calibration curve (Fig. 1). Means and standard deviations were calculated from three biological repeats for each strain. *, *P* < 0.001.

We used PHL2- and CPP-PHL2-tagged *C. albicans* strains to identify chemical agents that interfere with the normal regulation of intracellular pH. The NIH Clinical Collection (NCC) of 700 compounds was screened with the two strains at a final concentration of 10 µM by continuously monitoring the I_395_/I_470_ ratio for 24 h. A total of 155 compounds were identified that altered the I_395_/I_470_ ratio profile of either or both probes. This very high hit rate (22%) was likely the result of many drugs directly or indirectly affecting intracellular pH homeostasis through effects on growth kinetics, metabolic perturbation, and/or induction of stress responses. A large majority of these compounds (61%) only affected the vacuolar ratio, with 10% only affecting the cytosolic pH profile. Among the drugs that affected both PHL2-derived profiles were Benzbromarone and diflunisal, which we previously identified as potential vacuole-disrupting agents (23). The NSAID (nonsteroidal anti-inflammatory drug) diflunisal, which we previously reported induces abnormal vacuolar morphology in *C. albicans*, was identified from our screen as causing transient vacuolar acidification (Fig. 5A, ~9 to 10 h). Diflunisal treatment also resulted in a slightly more acidic cytoplasm upon entry into stationary phase. Endpoint measurements at 24 h confirmed that diflunisal reduced both vacuolar and cytoplasmic pH, although only the cytoplasmic effect was statistically significant at this time point (Fig. 5A, bottom panel). Benzbromarone, a benzopyran derivative, has also been identified to cause cytoplasmic acidification in the model yeast *Saccharomyces cerevisiae* (24). Benzbromarone caused a delay in cytoplasmic acidification compared to the wild type; however, this coincided with a slight delay in growth kinetics. In addition, Benzbromarone-treated cells had a small reduction in the cytoplasmic I_395_/I_470_ ratio at later time points of the assay, indicating a slightly more acidic cytoplasm as the cells entered stationary phase (Fig. 5B). Benzbromarone did, however, greatly disturb the vacuolar pH profile, causing a transient but pronounced acidification between 8 and 11 h of treatment, followed by an apparent loss of acidification. A follow-up experiment using 50 µM Benzbromarone with endpoint measurements after 24 h of growth confirmed these findings (Fig. 5B, bottom panel). These data indicate that Benzbromarone and diflunisal as well as a large number of other medications can directly or indirectly affect intracellular pH homeostasis in *C. albicans*.

### The azole antifungals induce transient acidification of the *C. albicans* vacuole.

**FIG 6:**
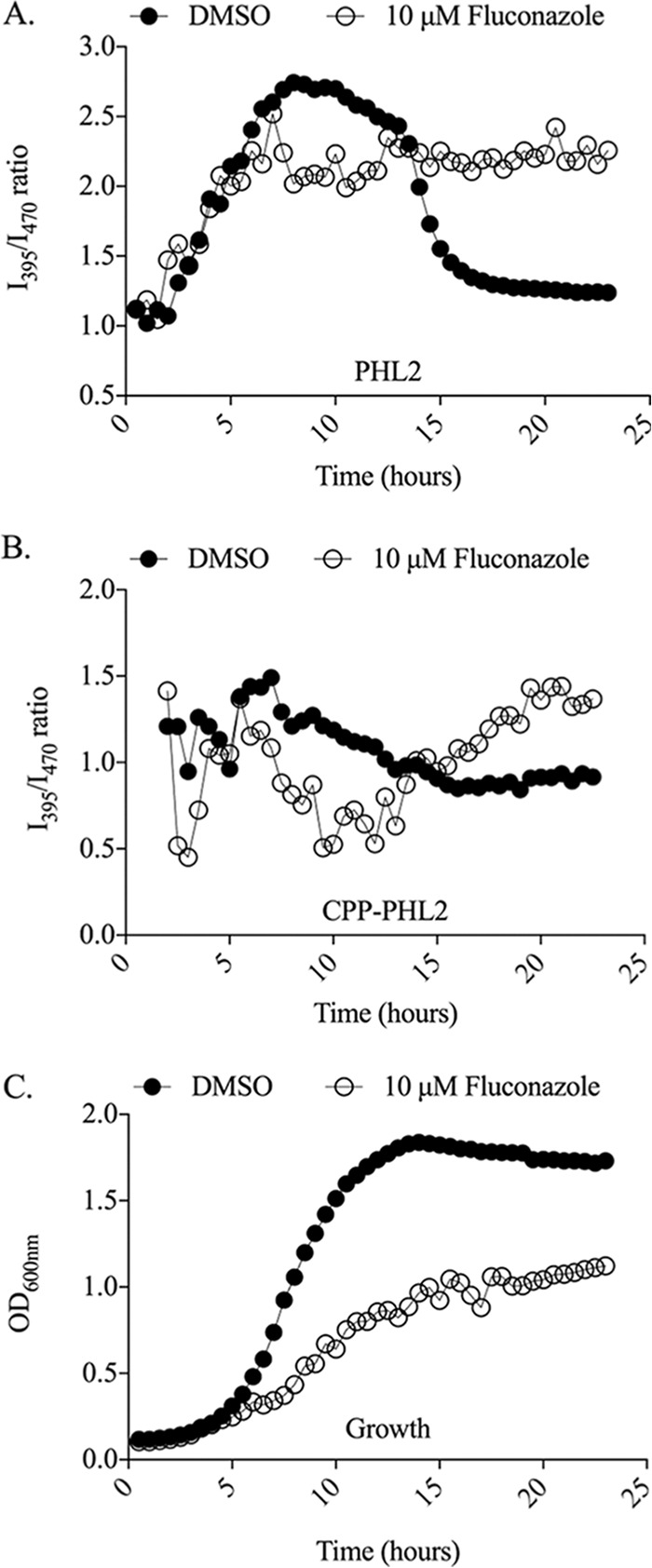
Fluconazole dysregulates cytoplasmic and vacuolar pH homeostasis in *C. albicans***.** CAI4 cells expressing PHL2 or CPP-PHL2 were grown in minimal medium at 35°C for 24 h in the presence of 10 mM fluconazole (open circles) or an equivalent amount of DMSO solvent (full circles). Fluorescence intensity from cytoplasmic PHL2 (A) or vacuolar CPP-PHL2 (B) and the OD_600_ (C) were measured every 30 min, and I_395_/I_470_ ratios were calculated for each time point, as described for Fig. 3.

Among the compounds identified as affecting both cytoplasmic and vacuolar pH, one-fourth affected growth greatly. These included fluconazole and several other azole antifungals. The azole antifungals have been proposed to act in part through inhibition of the V-ATPase complex, which is responsible for vacuolar acidification (25). We therefore tested the effect of fluconazole upon both cytoplasmic and vacuolar pH by using our PHL2 tools. Treatment of *C. albicans* with fluconazole caused an increase in intravacuolar pH (Fig. S3A), which was detected by an increase in the I_395_/I_470_ ratio with our CPP-PHL2 probe when endpoint measurements were taken after 24 h of treatment. These data were in agreement with previous reports that fluconazole causes loss of vacuolar acidification (4, 25). The cytoplasmic PHL2 probe revealed that fluconazole also caused a concurrent increase in cytoplasmic pH, which had not been previously reported (Fig. S3A). However, time course analysis revealed more complex and dynamic intracellular pH responses of *C. albicans* cells to fluconazole. The drop of the cytoplasmic PHL2 ratio observed upon entry into stationary phase in the control cultures was not observed upon fluconazole treatment, and hence pH remained at a much higher level than in the dimethyl sulfoxide (DMSO) control (Fig. 6A). Vacuolar pH also differed greatly in treated cells, with a period of lower ratios (indicative of lower pH) occurring between 8 and 12 h of treatment, followed by an elevated ratio (indicative of a higher pH) above that of the DMSO control (Fig. 6B). This transient vacuolar acidification in response to fluconazole may be an adaptive response to antifungal stress. Yet, cells exposed to fluconazole for 24 h failed to acidify their cytoplasm or display a loss of vacuolar acidity. However, these changes coincided with significant growth inhibition (Fig. 6C), and it remains unclear if the observed loss of acidification of the cytoplasm following fluconazole treatment is a direct consequence of the drug’s mode of action or merely a secondary consequence of altered growth phases and kinetics. Either way, the cytoplasmic pH response profile of an azole-resistant clinical isolate, TW17 (26), in the presence of 10 µg/ml fluconazole was similar to the response profile of SC5314 in the absence of the drug (Fig. S4). A second clinical isolate (TW1) (26) that exhibits reduced fluconazole sensitivity under the experimental conditions used produced growth and cytoplasmic profiles that were intermediate between TW17 and SC5314. This further supports the notion that the impact of fluconazoles on intracellular pH homeostasis is perhaps unsurprisingly inseparable from their impact on fungal growth. Similar pH response profiles were observed when other azole antifungals were used in place of fluconazole (Fig. S3B).

10.1128/mSphere.00276-17.3 FIG S3  pH shifts upon azole treatment in *C. albicans*. (A) Cytoplasmic and vacuolar pH shifts as an endpoint measurement following fluconazole treatment. Cells expressing PHL2 (full circles) or CPP-PHL2 (open circles) were grown in minimal medium, and fluconazole was added at the indicated concentrations. Cultures of 1 ml were grown at 35°C for 24 h. Fluorescence was measured by flow cytometry, with a minimum of 20,000 events under each conditions. Two independent experiments, each including 3 biological repeats per strain and per conditions were used for the analysis. (B) pH profiles in response to imidazoles. Cells were grown in minimal medium at 35°C for 24 h in the presence of 10 mM of compounds or 1% DMSO in a final volume of 100 ml in a 96-well plate format. Fluorescence at 395 and 470 nm (emission at 509 nm) from cytoplasmic PHL2 (left) or vacuolar CPP-PHL2 (middle) and absorbance at 600 nm (right) were acquired every 30 min. I_395_/I_470_ ratios were calculated at each time point after subtraction of the background fluorescence from cells expressing the empty vector. Download FIG S3, PDF file, 0.1 MB. Copyright © 2017 Tournu et al. 2017 Tournu et al. This content is distributed under the terms of the Creative Commons Attribution 4.0 International license.

10.1128/mSphere.00276-17.4 FIG S4  pH shifts upon fluconazole treatment in three clinical isolates with a wide range of susceptibility to the azole. Cells were grown in minimal medium at 35°C for 24 h in the presence of 10 μg/ml of fluconazole or 1% DMSO in a final volume of 100 ml in a 96-well plate format. Fluorescence levels at 395 and 470 nm (emission at 509 nm) from cytoplasmic PHL2 (empty circles) and absorbance at 600 nm (full squares) were acquired every 30 min from wild-type strain SC5315 (top), TW1 strain (middle), and TW17 strain (bottom). I_395_/I_470_ ratios were calculated at each time point (after subtraction of the background fluorescence, i.e., that of cells expressing the empty vector). A red arrow indicates the entry into growth arrest, which coincided with the sharp drop of cytoplasmic pH. Download FIG S4, PDF file, 0.2 MB. Copyright © 2017 Tournu et al. 2017 Tournu et al. This content is distributed under the terms of the Creative Commons Attribution 4.0 International license.

While both the cytoplasm and vacuole appeared to be less acidic following longer periods of azole treatment, it was notable that CPP-PHL2 I_395_/I_470_ ratios remained significantly below those of the PHL2 probe throughout the growth cycle. This clearly indicated that the vacuole lumen remains relatively acidic compared to the cytoplasm in fluconazole-treated cells.

## DISCUSSION

Adaptation of PHL2 for use in *C. albicans* has provided a very bright and robust indicator of intracellular pH in this important fungal pathogen. The dramatically enhanced sensitivity of PHL2 detection makes it well suited for *in situ* measurements of cellular pH without the need for addition of reagents, buffer changes, or other manipulations. As we have demonstrated, these properties facilitate noninvasive longitudinal analysis of cell-associated pH to monitor physiological responses in real time. It is also now possible to monitor pH fluxes in total cell populations in a convenient 96-well plate format, or in individual cells using a flow cytometry-based protocol or potentially advanced microscopic techniques. The ability to measure changes in individual cells is an important advance, as it will enable the identification of different subpopulations of cells within a culture that may respond differently to a given stimulus or growth condition.

Together, the tools described herein provide a convenient and sensitive means to measure cytoplasmic, vacuolar, and cell surface pH in the pathogen *C. albicans*. Targeting any reporter protein to the proteolytically active interior of the fungal vacuole poses significant challenges due to the high potential for its degradation. However, by using the prepropeptide from *C. albicans* carboxypeptidase Y, we successfully targeted PHL2 to the vacuole lumen, where it is both functional and sufficiently stable to provide a sensitive marker of vacuolar pH. Indeed, almost identical pH-I_395_/I_475_ ratio-response curves were produced with cytoplasmic, vacuolar, and cell surface versions of PHL2. Nonetheless, as with any other dye- or PHL-based experiment, for optimal pH measurements we recommend establishing a calibration curve for each individual growth condition to avoid variations between experiments. However, when conducting a comparative analysis (for example, between strains or chemical treatment groups), relative pH can be conveniently expressed as the I_395_/I_470_ ratio.

One potential limitation of the PHL2-based probes described herein was revealed when we used the PHL2-Pga59p fusion, which lost fluorescence following prolonged culture in medium with a pH of <6. Nevertheless, transient acidification can be accurately monitored, as established with the calibration buffers and our experiments in which the cell surface PHL2-Pga59p probe retained ratiometric function for up to 4 h following transfer to acidic medium. The PHL2-Pga59p fusion is also functional over a substantial range of the pH conditions that *C. albicans* cells are likely to encounter within their mammalian host. Our data also suggest that vacuolar pH in *C. albicans* cells grown under standard laboratory conditions is not acidic enough to impair the functionality of the pH probe. Accordingly, we anticipate that the ratiometric PHL2-based probes will provide useful tools to investigate *C. albicans* physiological responses to external factors and antifungal drugs and also interactions with host cells. For example, the three PHL2 probes could be used to characterize the interaction of *C. albicans* with phagocytic cells through a flow cytometry-based analysis. Such studies would be highly significant, as recent reports have established that *C. albicans* is able to neutralize the phagolysosome of macrophages, which leads to pyroptosis (27).

Time course measurements revealed a surprisingly large fluctuation in cytoplasmic pH throughout the growth cycle of *C. albicans*. Differences of 2 pH units were observed between exponentially growing and stationary-phase cells. This is consistent with previous reports for *S. cerevisiae* (21) and *Candida glabrata* (3), that showed that cytoplasmic pH is a function of growth rate. Delay or loss of cytoplasmic pH fluctuation was associated with a delay in growth arrest or slow growth, as demonstrated with cells treated with Benzbromarone or azoles, respectively. How much cell viability influences the pH profiles of cells treated with azoles remains to be investigated. In contrast, vacuolar pH fluctuated by less than 1 pH unit through the growth cycle. Interestingly, among the few clinical isolates used in this study, we identified one, DR17, which seems to be deficient in vacuolar acidification. This is surprising, as several V-ATPase-deficient gene deletion mutants of *C. albicans* have been reported to have reduced pathogenicity in a mouse model of disseminated candidiasis (9, 10). However, loss of V-ATPase function may not have such a profound impact upon the ability of *C. albicans* to colonize or cause disease at one or more of the mucosal membranes that are its primary habitat. On a broader note, it is unclear if variation in V-ATPase activity or regulation between clinical isolates is sufficient to influence *in vivo* fitness or to confer a selective advantage within a particular host niche. Addressing such questions will require significant further investigation but can in part be facilitated through the rapid, sensitive, convenient, and real-time measurements made possible with our new PHL2 tools.

The chemical screen using PHL2 and CPP-PHL2 provided several interesting insights. First, the high hit rate with a chemical library enriched for approved medications suggests that, unsurprisingly, intracellular pH is directly or indirectly regulated by a large number of physiological processes, and this is likely to include fungal growth rate. That said, it is possible to uncouple regulation of intracellular pH and growth, as drugs such as simvastatin and phenelzine sulfate were identified that affect either cytoplasmic or vacuolar pH without affecting growth rate at the concentration tested (data not shown). These compounds may provide useful chemical probes to investigate the mechanisms of intracellular pH homeostasis. Finally, while our results were consistent with previous reports that prolonged exposure to the azole antifungals alkalinizes the *C. albicans* vacuole, our results also provided a new context, by revealing the simultaneous alkalization of the cytoplasm. This indicates that in fact the *C. albicans* vacuole remains relatively acidic compared with the cytoplasm following azole treatment.

## MATERIALS AND METHODS

### Growth conditions.

*C. albicans* was routinely grown on YPD agar (2% dextrose, 2% peptone, 1% yeast extract) plates at 30°C. Selection of *C. albicans* transformants was carried out on minimal YNB medium (6.75 g/liter yeast nitrogen base without amino acids, 2% dextrose, 2% Bacto agar) supplemented with the appropriate auxotrophic requirements or 50 μg/ml uridine. For transformation of prototrophic clinical isolates with the *SAT1* marker, selection was carried out on YPD agar plates containing 200 μg/ml nourseothricin (ClonNat; Gold Biotechnology).

### Plasmid construction.

Plasmids pLUX (28), pKE1 (29), pKE1-CPP (12), and pDUP3 (22) have been previously described. All oligonucleotides used in this study are listed in Table S1. For construction of plasmid pKE4, the *TEF1* promoter was amplified from SC5314 genomic DNA (gDNA) by using the primer pair TEF1prF-KpnI and TEF1prR-SalI and cloned between the KpnI and SalI sites of pKE1 to replace the *ACT1* promoter. The coding sequence of PHL2 (7) was optimized for efficient translation in *C. albicans* with the Optimizer online tool (Table S1) (30; http://genomes.urv.es/OPTIMIZER/), using the combined codon biases of the highly expressed genes *RPL29*, *RPL32*, *RPL39*, *ACT1*, and *ENO1*, and synthesized as a gBlock (Integrated DNA Technologies, Inc.). Flanking sequences containing upstream EagI and SalI sites and a downstream MluI site as well as primer binding sites for PCR amplification were included. PHL2 was amplified from the synthetic template using primers CDR1DETF and CDR1DETR, digested with SalI and MluI, and cloned between the same sites of pKE4, to yield pKE4-PHL2. The PHL2 coding sequence was digested with EagI and MluI and cloned between the same sites of pKE1-CPP to produce pKE1-CPP-PHL2.

PHL2 was then inserted within the *PGA59* coding sequence, downstream of the signal peptide sequence (16). *PGA59* (codons 20 to 113) was amplified from SC5314 genomic DNA with primers PGA59-EagI-F and PGA59-MluI-R and cloned into pKE1 between the EagI and MluI sites to create pKE1-PGA59. PHL2 was then amplified with primers SP-PHL2-SalI-F and PHL2-EagI-R, cut with SalI and EagI, and cloned between the same sites of pKE1-PGA59 to create pKE1-SP-PHL2-PGA59.

To facilitate expression of the pHluorin probes in clinical isolates, the PHL2 and CPP-PHL2 expression cassettes, including promoter and terminator regions, were amplified using primer pairs TEF1prom-ClaI-F/ADH1term-SpeI-R and ACT1prom-ClaI-F/ADH1term-SpeI-R, using pKE4-PHL2 and pKE1-CPP-PHL2, respectively, as the templates. Each product was cloned between the ClaI and SpeI sites of pDUP3. This generated vectors pDUP3-PHL2 (*TEF1* promoter) and pDUP3-CPP-PHL2 (*ACT1* promoter).

### *C. albicans* strains.

Strain SC5314 has been previously described (31), and CAI4 (32) was kindly provided by William Fonzi (Georgetown University). TW1 and TW17 were described previously (26). The DR isolates DR13, DR17/DR18, and DR37/DR38 were chosen because they carry homozygote SNPs in *PMA1*, *VMA13*/*22*, and *STV1*, respectively (D. Rogers, personal communication). *C. albicans* was transformed with DNA constructs by using the lithium acetate procedure (33). The *vph1Δ/Δ* gene deletion strain was constructed by the PCR-based approach described by Wilson et al. (34), using the *ura3Δ/Δ his1Δ/Δ arg4Δ/Δ* triple deletion strain BWP17 (kindly provided by Aaron Mitchell, Carnegie Mellon University) that has been described previously (35).

The following expression vectors were introduced into *ura3*^−^ recipient strains following digestion with NheI to target integration into (and reconstitute) the *URA3* loci (36): pKE1-CPP-PHL2, pKE4-PHL2, and pKE1-SP-PHL2-PGA59. Correct integration and thus reconstitution of the *URA3* loci was confirmed by PCR with primers LUXINTDETF and -R.

For transformation of prototrophic strains, pDUP3-PHL2 and pDUP3-CPP-PHL2 vectors were cut with NgoMIV and transformed as described above before plating on YPD plus nourseothricin plates. Correct integration at the NEUT5L locus was confirmed by PCR using primers NAT1-3118 and pDUP3-4969, as described by Gerami-Nejad et al. (22).

### Confocal microscopy.

Intracellular localization of PHL2 and CPP-PHL2 was determined by fluorescence microscopy with a Zeiss LSM 710 laser scanning confocal microscope. When required, *C. albicans* cells were prelabeled with the lipophilic dye FM4-64 to label the vacuolar membrane, as previously described (37). The laser wavelengths used were 488 nm and 546 nm, respectively, for pHluorin and FM4-64. The filters had bandpass ranges of 493 to 594 nm and 595 to 758 nm, respectively. FM4-64 intensity was enhanced in the merge images in order to highlight its localization over the PHL2 fluorescence.

### pH calibration of pHluorin2 probes.

The bimodal spectrum of *Candida* PHL2 was obtained across a range of excitation wavelengths from 370 to 490 nm. Calibration of the pHluorin2 probes was performed as previously described in the model yeast *S. cerevisiae* (38, 39). In brief, calibration buffers containing 50 mM morpholineethanesulfonic acid, 50 mM HEPES, 50 mM KCl, 50 mM NaCl, 200 mM ammonium acetate, 10 mM NaN_3_, 10 mM 2-deoxyglucose were adjusted to defined pHs with NaOH or HCl. The ratiometric (I_395nm_/I_470nm_) calibration curves were obtained across a range of defined pHs, from pH 5 to pH 7.5. Cells grown in YNB medium were resuspended into each calibration buffer, and 200-μl samples were dispensed into 96-well plates. The resulting curves were used to generate a third-degree polynomial regression equation, which in turn was used to estimate the pH of the samples tested.

### pHluorin-derived measurements.

Ninety-six-well plate pHluorin-derived measurements were obtained using a Cytation 5 plate reader (Bio-Tek Instruments, Inc.). Cells were routinely pelleted prior to measurement. For all calibration purposes and pH estimation, relative fluorescence units (RFU) were obtained with an emission filter at 509 nm (9-nm bandwidth) and excitation wavelengths of 395 nm and 470 nm (both with 9-nm bandwidth). Cells expressing the empty vectors were used as negative controls for background fluorescence. Background fluorescence measurements at 395 and 470 nm were subtracted from the RFU obtained from cells expressing the PHL2-based indicators, and the I_395_/I_470_ ratios were then calculated.

### Flow cytometry measurements.

For the fluconazole experiments, cells were grown in YNB minimal medium at 35°C. Up to 50,000 events were measured for each time point, and three biological replicates were analyzed using a NovoCyte flow cytometer (ACEA Biosciences, Inc.). Median fluorescence was obtained using a 488-nm fluorescein isothiocyanate blue laser and a 405-nm AmCyan violet laser, both with a 530/30 nm filter, to calculate the I_405_/I_488_ ratios after background subtraction (results for cells expressing empty vectors only).

### Chemical screening of the NIH Clinical Collection of compounds.

The NCC drug screening array containing 719 small molecules was purchased from the NIH Small Molecule Repository. Wild-type cells expressing cytoplasmic PHL2 or vacuolar CPP-PHL2 were grown overnight (O/N) in YPD, and cell suspensions were prepared at a 1/500 dilution of the O/N cultures in minimal medium. Samples of 100 μl of cell suspensions were dispensed in each well of 96-well plates containing the test compounds. For each plate, 8 DMSO-containing wells were used to generate an average DMSO profile. Compounds were tested at a 10 μM final concentration. Plates were incubated at 35°C in a Cytation 5 plate reader (Bio-Tek Instruments, Inc.) with continuous shaking. Fluorescence at 395 nm and 470 nm and absorbance at 600 nm were measured every 30 min for 24 h.
