# Supplementary figures and images for: In Vivo Indicators of Cytoplasmic, Vacuolar, and Extracellular pH Using pHluorin2 in Candida albicans
Source: mSphere. 2017 Jul 5;2(4):e00276-17. doi: 10.1128/mSphere.00276-17 (PMC5497024; doi:10.1128/mSphere.00276-17)

Supplemental Figure S1

A

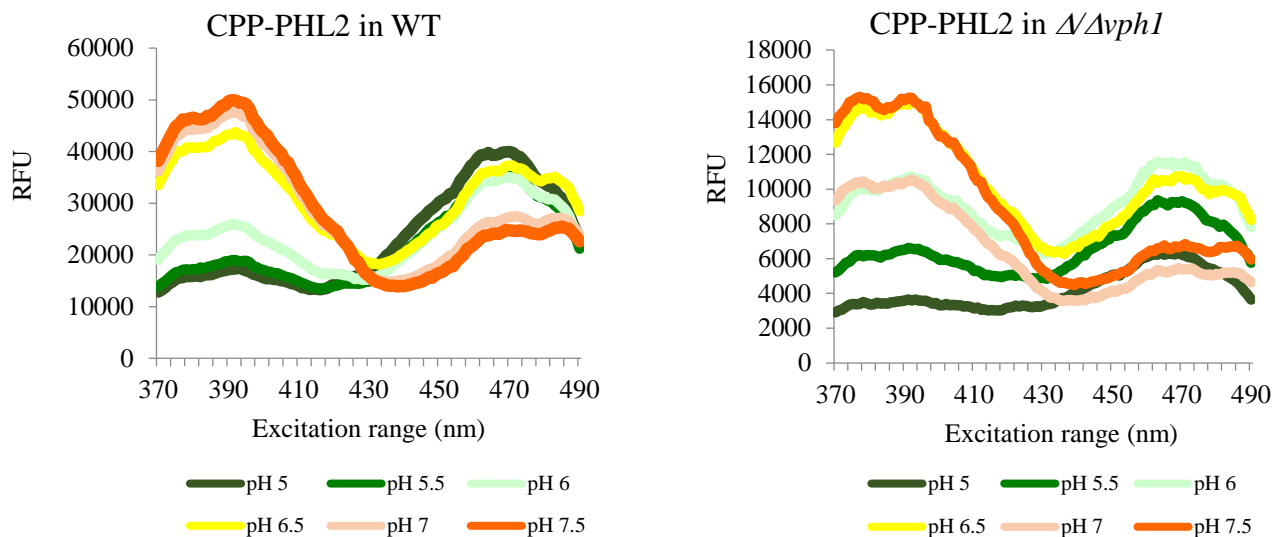

B

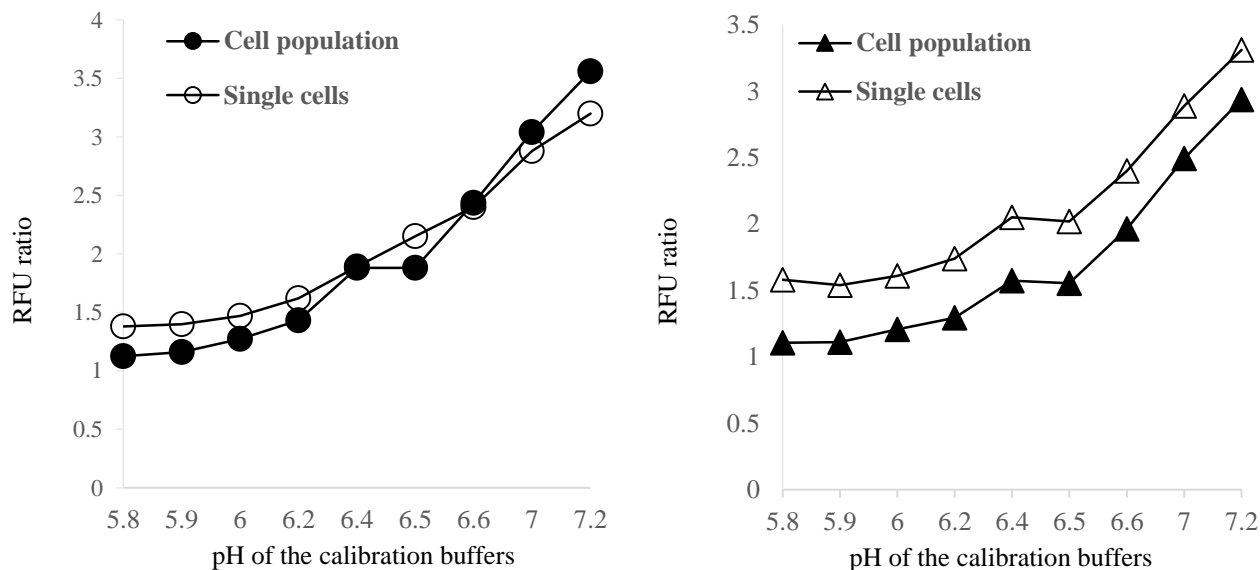

C

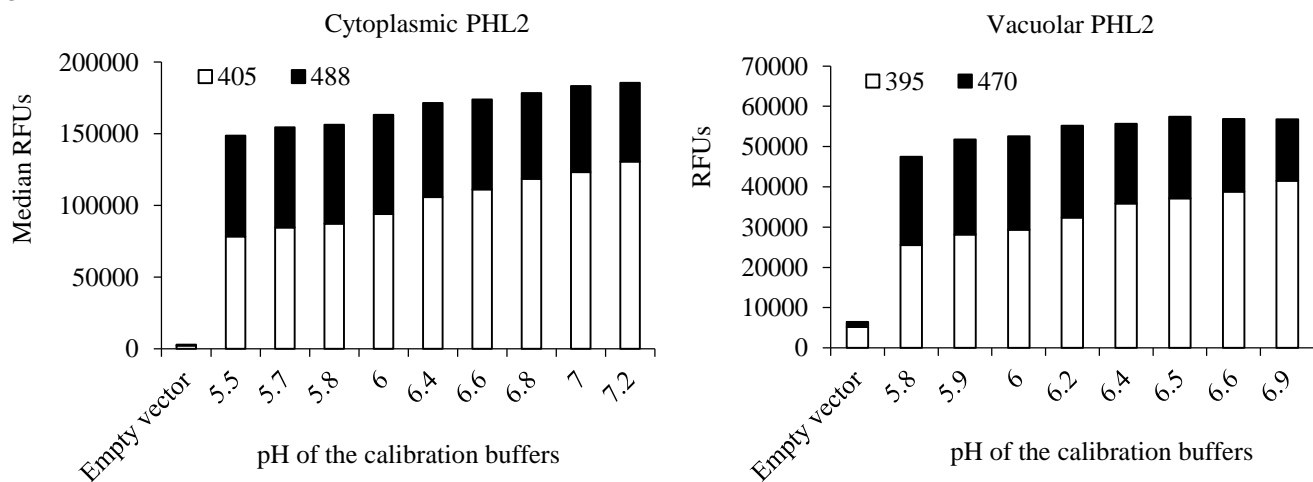

Supplement: FIG S1 [file sph004172315sf1.pdf]

Supplemental Figure S2

pH7

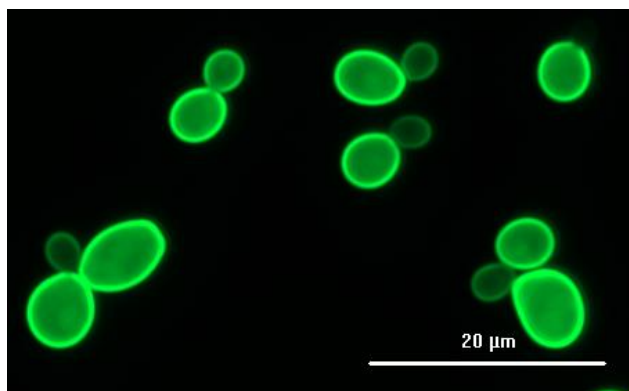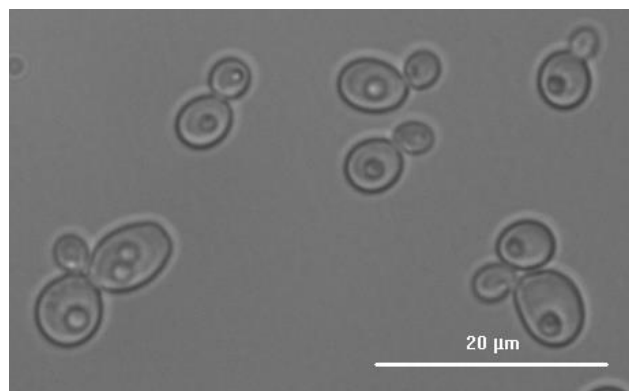

pH6.4

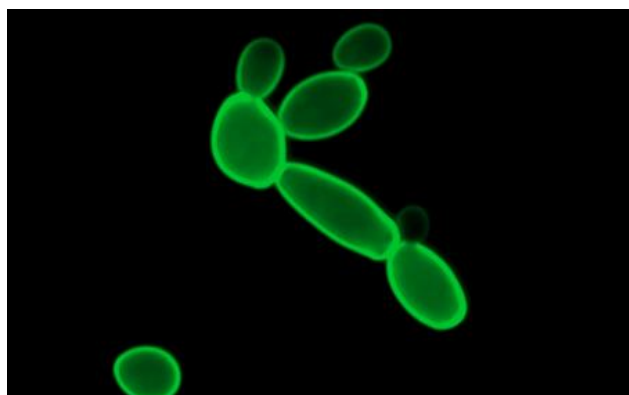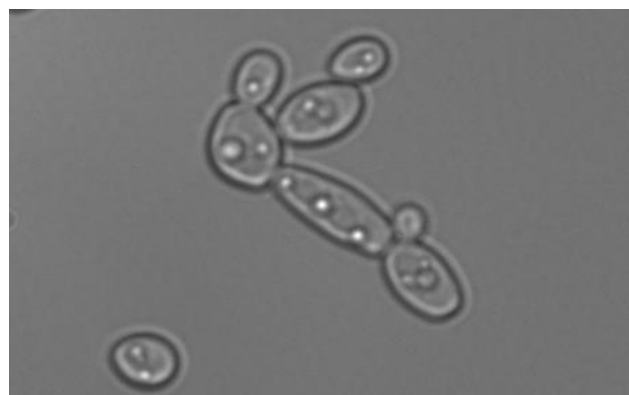

pH5.8

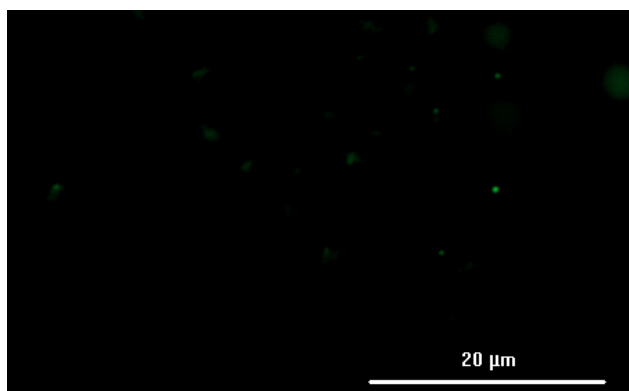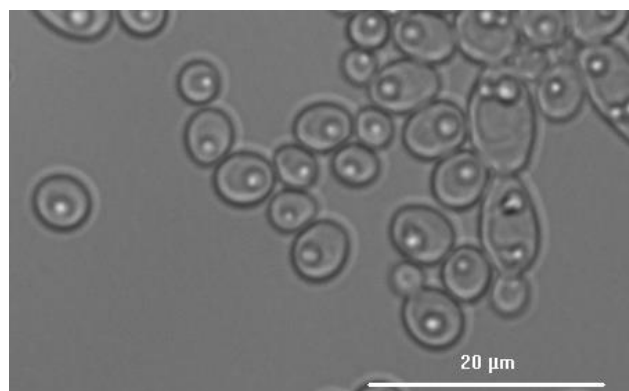

Supplement: FIG S2 [file sph004172315sf2.pdf]

A

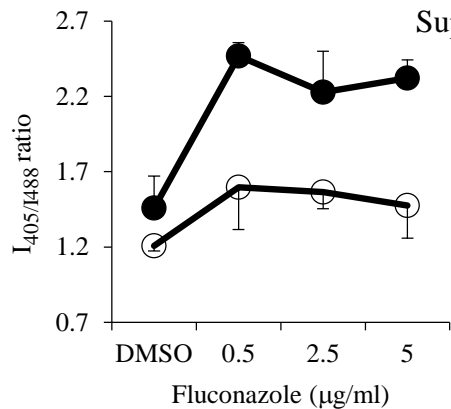

B

Cytoplasmic pH

Vacuolar pH

## Growth

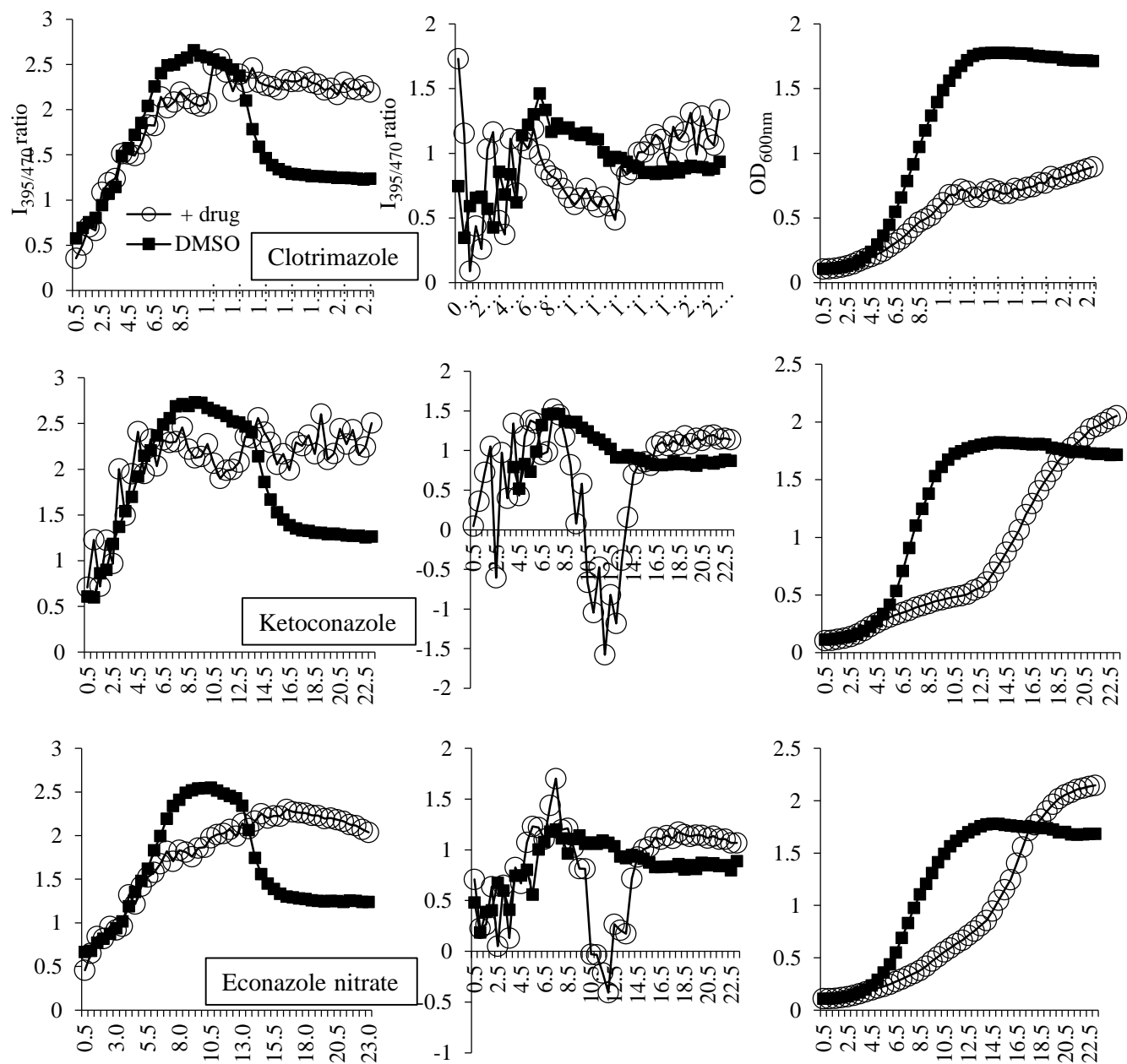

Supplement: FIG S3 [file sph004172315sf3.pdf]

Supplemental Figure S4

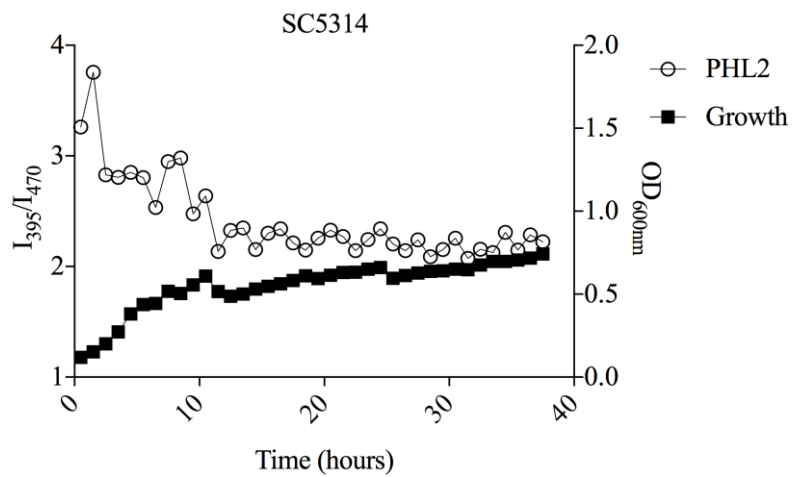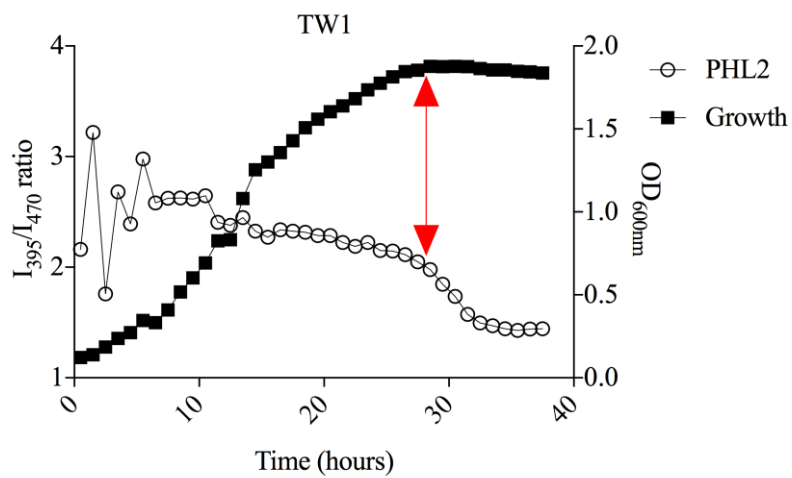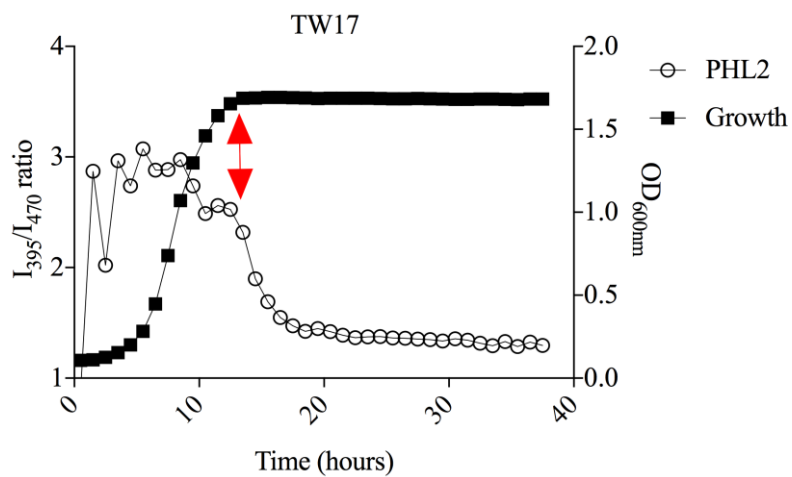

Supplement: FIG S4 [file sph004172315sf4.pdf]
