# Supplementary material for: In Vivo Indicators of Cytoplasmic, Vacuolar, and Extracellular pH Using pHluorin2 in Candida albicans
Source: mSphere. 2017 Jul 5;2(4):e00276-17. doi: 10.1128/mSphere.00276-17 (PMC5497024; doi:10.1128/mSphere.00276-17)
Supplement: TABLE S1 [file sph004172315st5.pdf]

Supplemental Table S1

Primer name	Sequence	Reference
TEF1pF-KpnI	TCAGGTACCTGCAAATCTGTTTGCTGATGG	(1)
TEF1pR-SalI	TCAGTCGACGATTGATTATGACTATAATGTG	(1)
CDR1DETF	TGCTGAAGCTTCTTTGAGTGG	This study
CDR1DETR	AGCAAAGAACATGGCAGCACC	This study
SP-PHL2-SalI-F	AGCGAGTCGACATGCAATTCTCATCCGCTATCATCTTATCC<br>GCTGTTGCTGGTTCCGCTTTAGCCACTTACATGTCTAAAGG<br>TGAAGAATTGTTCACTGGTG	This study
PHL2-EagI-R	AGCGACGGCCGTTTGTACAATTCATCCATACCGTG	This study
PGA59-EagI-F	AGCGACGGCCGGCTAACTCCACTGTCACTGACATTGC	This study
PGA59-MluI-R	AGCGAACGCGTTTACATGAAAGCACCCAAAGCCAACAAA<br>CCGG	This study
TEF1prom-ClaI-F	TGACAATCGATTGCAAATCTGTTTGCTGATGGAC	This study
ADH1term-SpeI	TGACAACTAGTGAAAACTTGAAACTTGAAAACACCG	This study
ACT1prom-ClaI-F	TGGACAATCGATCCAGCCTCGTTTATAATAAACTTAGTC	This study
LUXINTDETF	CTGACCTTTAGTCTTTCCTGC	(2)
LUXINTDETR	CAGTAGTACTTGTTGTTGTATCG	(2)
NAT1-3118	CCCAGATGCGAAGTTAAGTGCGCAG	(3)
pDUP3-4969	GGATTTAGTTCCATTATGG	(3)
C. albicans optimized PHL2	***ATG***TCTAAAGGTGAAGAATTGTTCACTGGTGTTGTTCCAA<br>TTTTGGTTGAATTGGATGGTGATGTTAACGGTCACAAATT<br>CTCTGTTTCTGGTGAAGGTGAAGGTGATGCTACTTACGGT<br>AAATTGACTTTGAAATTCATTTGTACTACTGGTAAATTGCC<br>AGTTCCATGGCCAACTTTGGTTACTACTTTGTCTTACGGTG<br>TTCAATGTTTCTCTAGATACCCAGATCACATGAAACAACA<br>CGATTTCTTCAAATCTGCTATGCCAGAAGGTTACGTTCAA<br>GAAAGAACTATTTTCTTCAAAGATGATGGTAACTACAAAA<br>CTAGAGCTGAAGTTAAATTCGAAGGTGATACTTTGGTTAA<br>CAGAATTGAATTGAAAGGTATTGATTTCAAAGAAGATGGT<br>AACATTTTGGGTCACAAATTGGAATACAACTACAACGAAC<br>ACTTGGTTTACATTATGGCTGATAAACAAAAAAACGGTAC<br>TAAAGCTATTTTCCAAGTTCACCACAACATTGAAGATGGT<br>TCTGTTCAATTGGCTGATCACTACCAACAAAACACTCCAA<br>TTGGTGATGGTCCAGTTTTGTTGCCAGATAACCACTACTTG<br>CACACTCAATCTGCTTTGTCTAAAGATCCAAACGAAAAAA<br>GAGATCACATGGTTTTGTTGGAATTCGTTACTGCTGCTGGT<br>ATTACTCACGGTATGGATGAATTGTACAAATAA	This study

Engineered restriction enzyme sites are underlined. The ATG codon is highlighted in bold and italics.

References:

1. Peters BM, Luna-Tapia A, Tournu H, Rybak JM, Rogers PD, Palmer GE. 2017. An azole-tolerant endosomal trafficking mutant of *Candida albicans* is susceptible to azole treatment in a mouse model of vaginal candidiasis. Antimicrob Agents Chemother 61:e00084-17. https://doi.org/10.1128/AAC.00084-17.
2. Ramón AM, Fonzi WA. 2003. Diverged binding specificity of Rim101p, the *Candida albicans* ortholog of PacC. Eukaryot Cell 2:718–728. https://doi.org/10.1128/EC.2.4.718-728.2003.
3. Gerami-Nejad M, Zacchi LF, McClellan M, Matter K, Berman J. 2013. Shuttle vectors for facile gap repair cloning and integration into a neutral locus in *Candida albicans*. Microbiology 159:565–579. https://doi.org/10.1099/mic.0.064097-0.
